# EEG-based neurodegenerative disease diagnosis: comparative analysis of conventional methods and deep learning models

**DOI:** 10.1038/s41598-025-00292-z

**Published:** 2025-05-07

**Authors:** B. R. Nayana, M. N. Pavithra, S. Chaitra, T. N. Bhuvana Mohini, Thompson Stephan, Vijay Mohan, Neha Agarwal

**Affiliations:** 1 Department of Computer Science and Engineering, M. S. Ramaiah University of Applied Sciences, Bangalore, Karnataka India; 2 Thumbay College of Management and AI in Healthcare, Gulf Medical University, Ajman, United Arab Emirates; 3 Department of Mechatronics, Manipal Institute of Technology, Manipal Academy of Higher Education, Manipal, Karnataka 576104 India; 4 School of Chemical Engineering, Yeungnam University, Gyeongsan, 38541 Republic of Korea

**Keywords:** Dementia, Deep learning, Decomposition, Feature extraction, Power spectral density, Spectrogram, Biomedical engineering, Neurological disorders, Machine learning

## Abstract

In the context of lifestyle changes, stress and other environmental factors have resulted in the sudden hike in dementia globally. This necessitates investigations with respect to every horizon of the due cause for it; further on, the diagnosis and treatments can be advanced with the aid of technology. This work attempts to conduct one such investigation on dementia diagnosis based on EEG signals. The implementation is carried out under three different verticals. Firstly, a conventional machine learning model was developed post-pre-processing, and feature extraction from the power spectral density was done using a Random Forest classifier. Second, 1D Convolutional Neural Networks models are developed, and pre-processed EEG signals are fed as input. Third, stacked spectrogram images are computed from decomposed EEG signals and are fed to 2D CNN models for classification. The investigations are performed on three different benchmark datasets. Across three datasets, the class labels include cognitively normal, frontotemporal dementia, mild cognitive impairment, and Alzheimer’s. The study offers a comparative evaluation across three distinct datasets, illustrating that deep learning models, particularly 1D and 2D CNNs, consistently outperform conventional methods in recognizing subtle EEG signal patterns linked to neurodegenerative conditions. For instance, in Dataset 1, the 2D CNN achieved the highest accuracy of 91.13%, surpassing the Random Forest model’s 84.78% accuracy. Nevertheless, the investigation also points out challenges in Dataset 3, indicating the necessity for further model optimization tailored to specific datasets. Statistical tests validate the findings. This study showcases a comparative investigation of the potential of deep learning models vs. conventional classifiers in clinical environments for the early diagnosis of dementia.

**Supplementary Information:**

The online version contains supplementary material available at 10.1038/s41598-025-00292-z.

## Introduction

Dementia encompasses various neurodegenerative disorders characterized by a progressive decline in cognitive abilities, affecting memory, thinking, behavior, and daily living skills. More than 55 million people worldwide are living with dementia, with nearly 10 million new cases reported each year. This number is expected to nearly double every 20 years, potentially reaching 78 million by 2030 and 139 million by 2050, particularly in low- and middle-income countries^1^. Alzheimer’s disease (AD) is the most common form of dementia, accounting for 60–70% of all cases^2^. As of 2024, roughly 6.9 million people in the United States over the age of 65 had lived with Alzheimer’s, and projections suggest that without any medical advances, this figure could swell to 13.8 million people by 2060^3^. Dementia is the seventh leading cause of death and is one of the most important causes of disability and dependence among older adults worldwide. The timely and appropriate diagnosis of dementia is essential for management and intervention. Electroencephalography (EEG) is a non-invasive method used to record the electrical activity in the brain^4^. It has also been suggested to be useful for diagnosing AD at its earliest stages. Deep Learning (DL) approaches to EEG signal processing in clinical contexts: Towards reliability and accuracy of diagnostic tools for detecting AD using EEG-based clinical decision support systems^5^. Several works utilize ML and DL techniques on EEG data for classifying and detecting Alzheimer’s. In the review of the literature published between 2013 and 2023, typical ML and DL applications for AD diagnosis via EEG are summarised, revealing an increasing interest in this field of research^6^. New analytical techniques can identify subtle patterns in the EEG signals that are indicative of varying states of dementia, helping to enable earlier diagnosis and also tailored treatment plans. Various DL models for EEG-based AD classification have been proposed in recent studies. A completely novel end-to-end DL model, namely, CAUEEG, has been proposed for the separation of AD patients from normal subjects and MCI patients based on EEG data^7^. Another study proposes a technique for tracking and diagnosing AD based on sophisticated EEG signal processing, illustrating DL’s promise in this arena^8^. The integration of DL models in combination with EEG analysis has a number of advantages over traditional methods^9^. DL models, including 1D and 2D CNNs, can automatically learn complicated features from either raw EEG signals or EEG features obtained using conventional methods, which may enhance the prediction accuracy of neurodegenerative diseases. For example, in a study comparing three datasets, classifying cognitive states with 2D CNNs achieved higher accuracy rates than traditional Random Forest (RF) classifiers. However, there are still some challenges in the application of EEG-based dementia detection using DL. Differences in EEG data between populations and recording conditions could impact model performance. A significant hurdle is the need for large, well-annotated datasets in order to train DL models. These models are subject to further research to improve their reliability and generalizability, including issues specific to the dataset from which they are derived. Overall, the global rising prevalence of dementia reinforces the necessity for early detection and intervention methods. EEG study, as well as ML & DL techniques used here, are data-driven methods that can be very useful and can improve the precision and time of dementia diagnosis. Ongoing research and development in this field will be essential for harnessing the full potential of these technologies in clinical practice, which will result in improved patient outcomes and more streamlined delivery of health care.

Among such developments, in the context of early detection of AD, EEG data has leveraged a significant potential. AD nonlinear analysis methods: numerous studies have decrypted EEG signals collected from AD patients and considered that EEG nonlinear analysis is possible for developing an early, rapid, and accurate diagnosis of AD. Miltiadous et al.^10^ calculated the PSD of time-windowed signals using the Welch method to classify between the AD, FT, and CN groups. They used different ML models that included lightgbm (optimized with Hyperopt), a Multilayer Perception (MLP) comprising one hidden layer with three neurons, RM, a Support Vector Machine (SVM) with a polynomial kernel, and k-Nearest Neighbors (kNN) (k = 3). Wang et al.^11^ performed PSD estimation based on the autoregressive Burg method with a sliding Hamming window and estimated coherence based on cross-spectral spectra for pairwise EEG channels. Statistical analysis was performed by one-way ANOVA^12^ using Bonferroni correction to evaluate differences between groups. Classification of AD patients versus controls was achieved by utilizing relative PSD and coherence in specific frequency bands. Göker et al.^13^ applied the multitaper method for feature extraction to compute PSD values of EEG signals across the 1–49 Hz frequency range, capturing critical frequency-domain information for AD detection. They used ensemble learning classifiers, including AdaboostM1, Total Boost, Gentle Boost, Logit Boost, Robust Boost, and Bagging, to effectively differentiate the EEG signals of AD patients from those of healthy individuals. In recent years, DL approaches have achieved significant success in diagnosing AD. Compared to traditional ML methods, DL models offer higher accuracy and greater efficiency in detecting AD. Hong et al.^14^ propose a predictive model that uses Long Short Term Memory (LSTM), which is a Recurrent Neural Network (RNN) that predicts MCI from AD. Al Shehri et al.^15^ developed a DL-based solution using DenseNet-169 and ResNet-50 CNN architectures for the diagnosis and classification of AD. Datta et al.^16^ used a DL model, specifically a modified 1D CNN, to classify EEG signals into three classes: normal, MCI, and moderate dementia. The model comprises 11 layers, including Conv1D, Batch Normalization, LeakyReLU, MaxPool1D, Dropout, Average Pooling1D, Global Average Pooling1D, and Dense layers. Deshmukh et al.^17^ used Discrete Wavelet Transform (DWT) and statistical parameters (mean, variance, skewness, kurtosis, and standard deviation) for feature extraction. The classification was performed using SVM and KNN, while dataset size limitations were addressed through data augmentation with the CTGAN architecture. Their CNN model for classification included an input layer, a convolutional layer, a pooling layer, an LSTM layer, and a dense layer. Dao et al.^18^ extracted x-velocity and y-velocity features from raw coordinates and timestamps and applied data augmentation using traditional methods and DoppelGANger. A 1D CNN, optimized through hyperparameter tuning, was utilized for sequential data processing. The model’s architecture was refined by adjusting convolutional layers, filters, filter sizes, and dropout. Supervised learning techniques were then applied to classify AD and Healthy Controls (HC) based on time series features. Tawhid et al.^19^ utilized stationary wavelet transformation to denoise signals, segmented them into smaller time frames, and extracted four frequency sub-bands. Spectrogram images were generated for each sub-band and the full frequency band, resulting in five image sets, which were classified using a 2D CNN. Their analysis of MCI datasets revealed that the 16–32 Hz sub-band had the most significant impact on detection, followed by the 4–8 Hz band. Nour et al.^20^ proposed a deep ensemble learning framework integrated with 2D CNNs to classify EEG signals for AD and HC subjects, employing five distinct 2D CNN models as internal classifiers. Şeker et al.^21^ introduced DWT-CNN, a practical AD detection tool that uses DWT to extract EEG sub-bands. By employing a Hann window for PSD estimation, they used a Conv2D architecture to classify spectrograms of EEG sub-bands, effectively enhancing AD diagnosis by capturing both temporal and frequency information. Table 1 presents a comparative analysis of AD cognitively normal (CN), frontotemporal dementia (FTD) classification methods.

**Table 1:** Comparative analysis of AD classification techniques.

References	Methodology	Dataset	Classes	Accuracy	
^10^		EEG Dataset	(AD, FTD, CN) 3	AD/CN	FTD/CN
	LightGBM			76.43%	72.43%
	SVM			73.14%	70.14%
	KNN			71.23%	67.34%
	MLP			73.12%	73.12%
	RF			77.01%	72.01%
^11^	Relative PSD	EEG Dataset	(AD, CN)^2^	88.5%	
	Normalized degree of functional connectivity obtained from coherence			82.9%	
	Combined feature			91.4%	
^13^	AdaboostM1	EEG Dataset	(AD, CN)^2^	93.04%	
	Total Boost			89.74%	
	Gentle Boost			93.04%	
	Logit Boost			93.04%	
	Robust Boost			87.18%	
	Bagging			83.52%	
^16^	1D CNN with 11 layers	EEG Dataset	(AD, MCI, CN) ^3^	97.31%	
^17^	1D CNN with Long Short-Term Memory	EEG Dataset	(AD, CN) ^2^	97.61%	
^18^	1DCNN with the use of GAN Augmentation data	Handwritten Dataset	(AD, CN) ^2^	87.04%	
^19^	Spectrogram images and 2D CNN	EEG Dataset	(MCI, CN) ^2^	99.03%	
^20^	DEL model used five different 2D CNN models	EEG Dataset	(AD, CN) ^2^	97.9%	
^21^	Discrete Wavelet Transform-2D CNN	EEG Dataset	(AD, CN) ^2^	100% for both EO and EC in the alpha stage band	

^*1,2,3^ refers to Datasets 1, 2, and 3 considered in the present work.

## Methodology

**Fig. 1:**
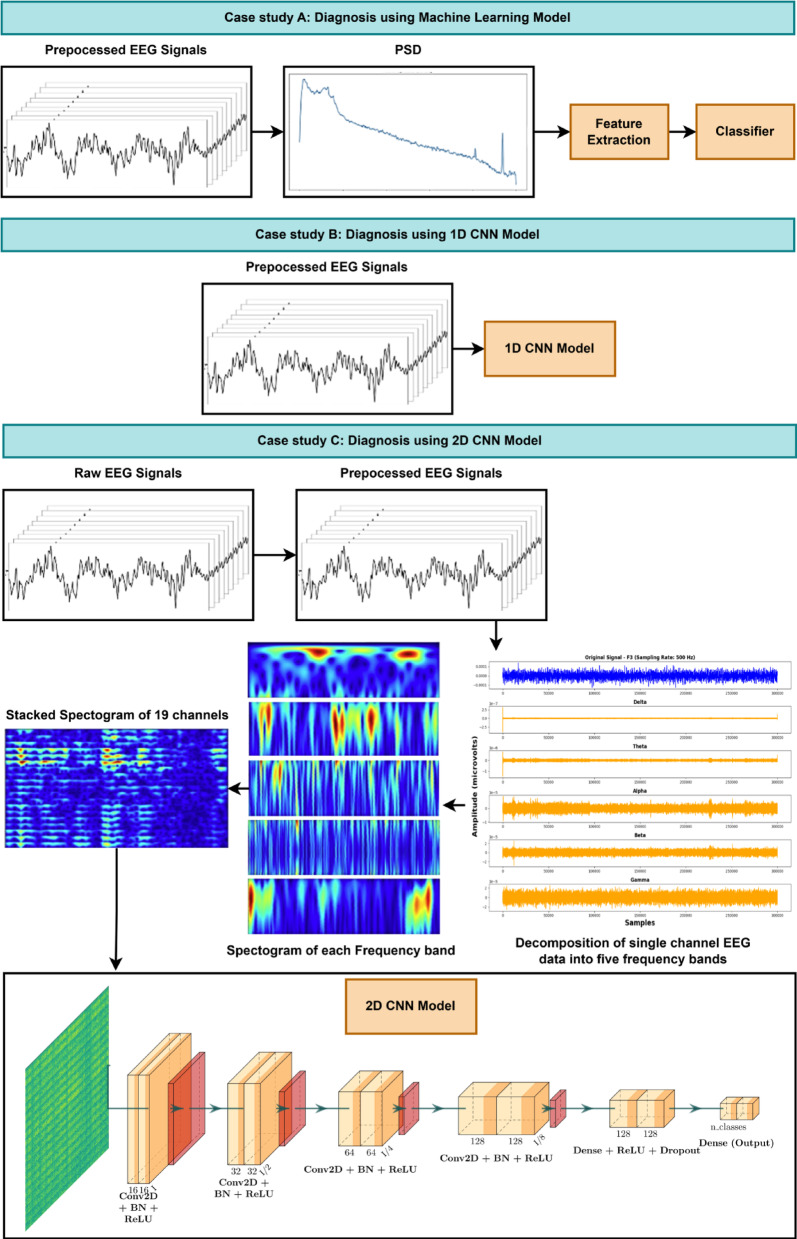
Block Diagram of EEG Data Analysis and Classification Pipe.

This study introduces a framework for diagnosing dementia from normal to Alzheimer’s based on EEG signals. The framework involves three diagnostic approaches: Firstly, a conventional classification approach, which involves feature extraction from Power Spectral Density (PSD) and classification using a classifier. Further, two approaches deal with DL models. The 1D CNN model utilizes pre-processed EEG signals, and the 2D CNN model is fed with stacked spectrogram images generated from decomposed EEG signals. The workflow of this methodology is presented in Fig. 1. The block diagram illustrates all three approaches to diagnosing dementia, which include CN, FTD, mild cognitive impairment (MCI), and AD using EEG signals. Case Study A involves an ML pipeline where frequency-domain features, mean, standard deviation, and variance are extracted from the PSD of pre-processed EEG signals. These features are then fed to the RF classifier. Case Study B employs a DL approach using a 1D CNN. Here, the pre-processed EEG signals are directly given as input into the 1D CNN model.

During this execution, 1D CNN models were developed to be unique for each dataset. In Case Study C, pre-processed EEG signals were decomposed into five standard frequency bands: Delta, Theta, Alpha, Beta, and Gamma, using a suitable wavelet transformation. Spectrogram images are generated for each frequency band of EEG data and are stacked across all 18 channels. This results in a collated single spectrogram image for a window segment of data considered for each band. These images are fed as input to 2D CNN models. Investigating the EEG data under different bands or decomposed signals aids in identifying the underlying patterns of dementia conditions. The 1D and 2D CNN architecture consists of multiple convolutional layers with Batch Normalization (BN) and ReLU activation, followed by dense layers with dropout for robust classification. This method leverages the spatial and frequency information within the EEG signals for accurate diagnosis. Together, these case studies showcase the progression from ML to advanced DL techniques for EEG-based diagnosis. While the study offers valuable insights, several limitations may affect the robustness and generalizability of its outcomes. Constraints in computational resources restricted the number of cross-validation folds and training epochs, potentially hindering optimal model performance. The imbalanced datasets presented additional challenges, as only basic techniques like up-sampling were applied, leaving more advanced approaches unexplored. Although pre-processing methods such as band-pass filtering and Independent Component Analysis (ICA) were utilized, the noise levels were high in certain datasets. Additionally, the heterogeneity across the datasets, two with three class labels and one with two, introduced inconsistencies that may have impacted the model’s generalization capabilities.

### Dataset description

In recent years, numerous research centers have accumulated significant amounts of medical data and made it publicly accessible. Public data plays a crucial role in researchers developing AI applications for AD research. Some of the most popular datasets are OpenNeuro (Miltiadous et al.)^10^, Isfahan University of Medical Sciences, Medical Image and Signal Processing Research Center (Tawhid et al.^19^, Neuroelectromagnetic Data Archive and Tools Resource (NEMAR), Mendeley Data Sedghizadeh et al. Scientific Data, Prado et al., October 2, 2023^22,23^, and Figshare (Cejnek et al.)^24^. In this work, Dataset A^25^ was selected based on OpenNeuro, a validated dataset with complete and relevant features for AD-related studies. EEG resting state data are available for 88 subjects, grouped into three categories (AD, *n* = 36), (FTD, *n* = 23), and (CN, *n* = 29). Dataset B is believed to originate from the Figshare website^24^. EEG data were obtained from 59 patients with moderate dementia, seven patients with MCI, and two controls. The EEG data is stored as *.mat files. Within this dataset, there are 33 AD patients and 4 MCI patients, whose data were collected at a sampling frequency of 256 Hz, and two normal controls with a sampling frequency of 128 Hz.

The selection of the Dataset C EEG Signals from Normal and MCI provides valuable insights into cognitive impairment research with EEG recordings from 27 participants, categorized into MCI with 11 individuals and CN with 16 individuals provided by Isfahan University of Medical Sciences, Medical Image and Signal Processing Research Center (Tawhid et al.^19^. Data were pre-processed through the application of a bandpass filter in order to keep the frequencies between 0.5 Hz and 32 Hz. This step was able to remove low-frequency drift and high-frequency noise to retain only relevant EEG-based frequency bands. Subsequently, ICA was performed to detect and remove artifacts (e.g., eye blinks and muscle activity) from the EEG data. ICA is a blind source separation method that can separate the EEG data into its independent components, allowing the identification and removal of artifacts such as eye blinks or muscle movements. In addition, for Dataset B, the EEG data for AD and MCI were acquired at a sampling rate of 256 Hz, and CN was acquired at 128 Hz. Up-sampling by linear interpolation of the CN data balances the subsequent analysis of all three classes, ensuring a smooth transition and preserving the data trend^26^.

### Experimental results

#### Case study A

PSD estimation using Welch’s method is applied to the pre-processed data to extract frequency-domain features, which are essential for further analysis. PSD describes how the power of a signal is distributed across different frequencies. Welch’s method was chosen for its robust ability to estimate PSD. Once the PSD is calculated, meaningful features are to be extracted from specific frequency bands (Delta-0.5–4 Hz, Theta-4–8 Hz, Alpha-8–13 Hz, Beta-13–25 Hz, Gamma-25–45 Hz) and then loaded into a suitable classifier model, as shown in Fig. 1. In this work, the RF classifier model is considered for its robustness in performance.

**Table 2:** ML model details for all datasets.

Aspect	Dataset - A	Dataset - B	Dataset - C
Segment Length	2000 (4 * 500 Hz)	768 (3 * 256 Hz)	768 (3 * 256 Hz)
Number of Segments	1672	72,124	322,449
Features Extracted	Mean, Standard Deviation, and Variance from each frequency band		
Overlap	50%	0%	0%
Frequency Bands	Delta, Theta, Alpha, Beta, Gamma	Delta, Theta, Alpha, Beta	Delta, Theta, Alpha, Beta
Number of Features per Segment	15 (3 features* 5 bands)	12 (3 features * 4 bands)	12 (3 features * 4 bands)
Exported Data per recording	(1672, 15)	(72124, 12)	(322449, 12)

For each band, statistical summaries (mean, median, standard deviation) of the PSD values within the frequency range are computed. These summaries provide concise information about the distribution and strength of brain activity across different frequency bands. The aspects considered for feature extraction with respect to each dataset, such as the length of each segment and overlap between segments, are defined to control the trade-off between frequency resolution and variance reduction in the estimated PSD presented in Table 2. All three conditions (AD, FTD, and CN) in Dataset A have higher power in the lower frequency ranges (0–50 Hz), which is typical of EEG signals that are dominated by the delta, theta, and alpha bands. Higher frequencies result in a decrease in power, and minute variations in the alpha and beta bands indicate neurophysiological alterations in each condition. These differences are crucial for finding biomarkers unique to a given condition. PSDs show a distinct progression of cognitive decline from normal to MCI to AD in Dataset B. While MCI exhibits intermediate fluctuations and normal conditions show stable brain activity, AD shows significant irregularities, particularly in higher frequencies, indicating neurodegeneration. This pattern emphasizes how brain function gradually deteriorates. The EEG channels of CN and MCI patients are compared in Dataset C, and both exhibit a decrease in power with frequency. PSD values are stable in the CN group, but MCI exhibits more variability and higher power at lower frequencies, suggesting changed brain activity. These distinctions aid in distinguishing between people in good health and those suffering from cognitive impairment^10^ Table 3 presents the pattern analysis of power distribution noted for each class in all 3 datasets. The supplementary material provides PSDs of all 19 channels pertaining to every class of each dataset.

**Table 3:** Spectral power distribution on PSD.

Feature	Dataset A	Dataset B	Dataset C
Delta Band (0.5–4 Hz)	AD: High power FTD: Increased CN: Low	AD: Very high MCI: Moderate CN: Low	MCI: Moderate increase CN: Low
Theta Band (4–8 Hz)	AD: Increased FTD: Slightly increased CN: Low	AD: Highly increased MCI: Increased CN: Moderate	MCI: Increased CN: Consistent
Alpha Band (8–12 Hz)	AD: Reduced FTD: Moderate CN: Balanced	AD: Highly reduced MCI: Slightly reduced CN: Consistent	MCI: Reduced CN: Normal
Beta Band (12–30 Hz)	AD: Minimal power FTD: Reduced CN: Moderate	AD: Almost absent MCI: Weak or absent CN: Moderate	MCI: Absent CN: Consistent
Gamma Band (30–45 Hz)	AD: Disrupted FTD: Moderate CN: Consistent	Not Included	Not included

**Fig. 2:**
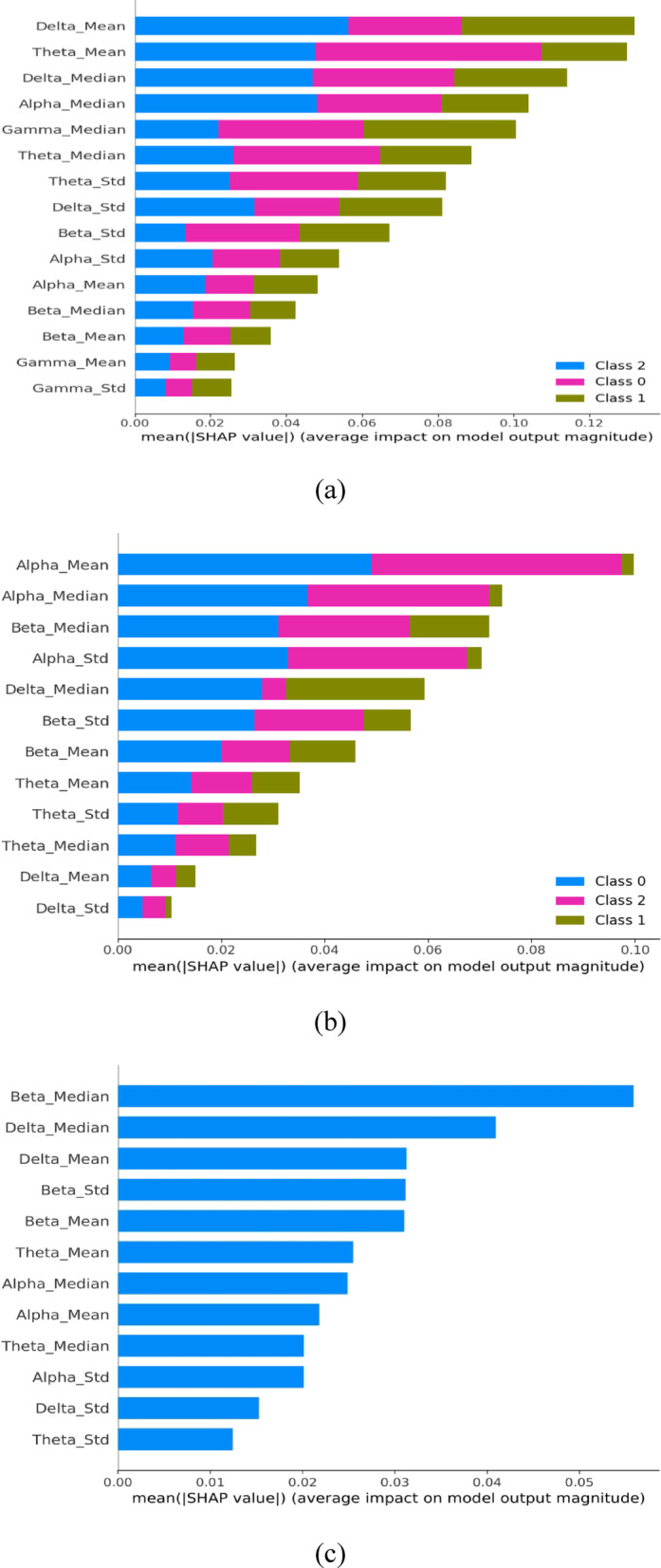
SHAP analysis of RF model for (**a**) Datasets A (**b**) Dataset B (**c**) Dataset C.

Analysing statistical measures such as mean, median, and standard deviation for Delta, Theta, Alpha, Beta, and Gamma bands while examining EEG signal characteristics across groups (AD, CN, and FTD). In the Delta and Theta bands, where clustering patterns aid in group differentiation, notable differences are seen. Smaller variations are seen in the alpha, beta, and gamma bands; the gamma band is the least noticeable, which may indicate less activity. Tighter clustering in CN suggests more regular brain activity. Delta and Theta bands show greater group differentiation, highlighting their relevance for identifying neurological conditions. Features such as Delta Mean, Delta Std, and Theta Mean exhibit clear separation, improving classification accuracy. Other features like Alpha Mean and Beta Median show some variation but maintain distinct clusters, suggesting high predictive potential. The scatter plots are provided in the supplementary material for reference. These descriptive analyses are supported by SHAP visualizations given in Fig. 2. In this work, an 80 − 20 split is considered, and the features are standardized to a mean of 0 and a standard deviation of 1. To enhance the model’s robustness, outliers in the training data were identified and removed using an Isolation Forest. The trained RF model was employed to predict labels on the test set. The model’s performance was assessed using metrics such as accuracy, precision, recall, F1 score, and specificity. These metrics offered insights into the classifier’s effectiveness and highlighted areas for potential improvement. The dataset A model scores in the range of 83.88-84.77%, and the dataset C model ranges between 77.3 and 77.6%, accuracy, precision, recall, and F1-score, respectively. Further, performance metric of RF for dataset B have accuracy of 90.15%, precision of 91.08%, recall of 90.15%, and F1-score of 87.73%.

As seen from the figures, for dataset A, delta mean and theta mean are the most significant features, whereas in dataset B, alpha mean and alpha median are prominent features. Also, the distribution of SHAP values across classes is more widespread in dataset A than in dataset B. Thus, it is perceived that the selection of crucial biomarkers for the diagnosis of neurodegenerative diseases using EEG can be guided by knowledge of which features are most important to the classification. SHAP analysis for dataset C does not reveal any specific pattern.

#### Case study B

**Fig. 3:**
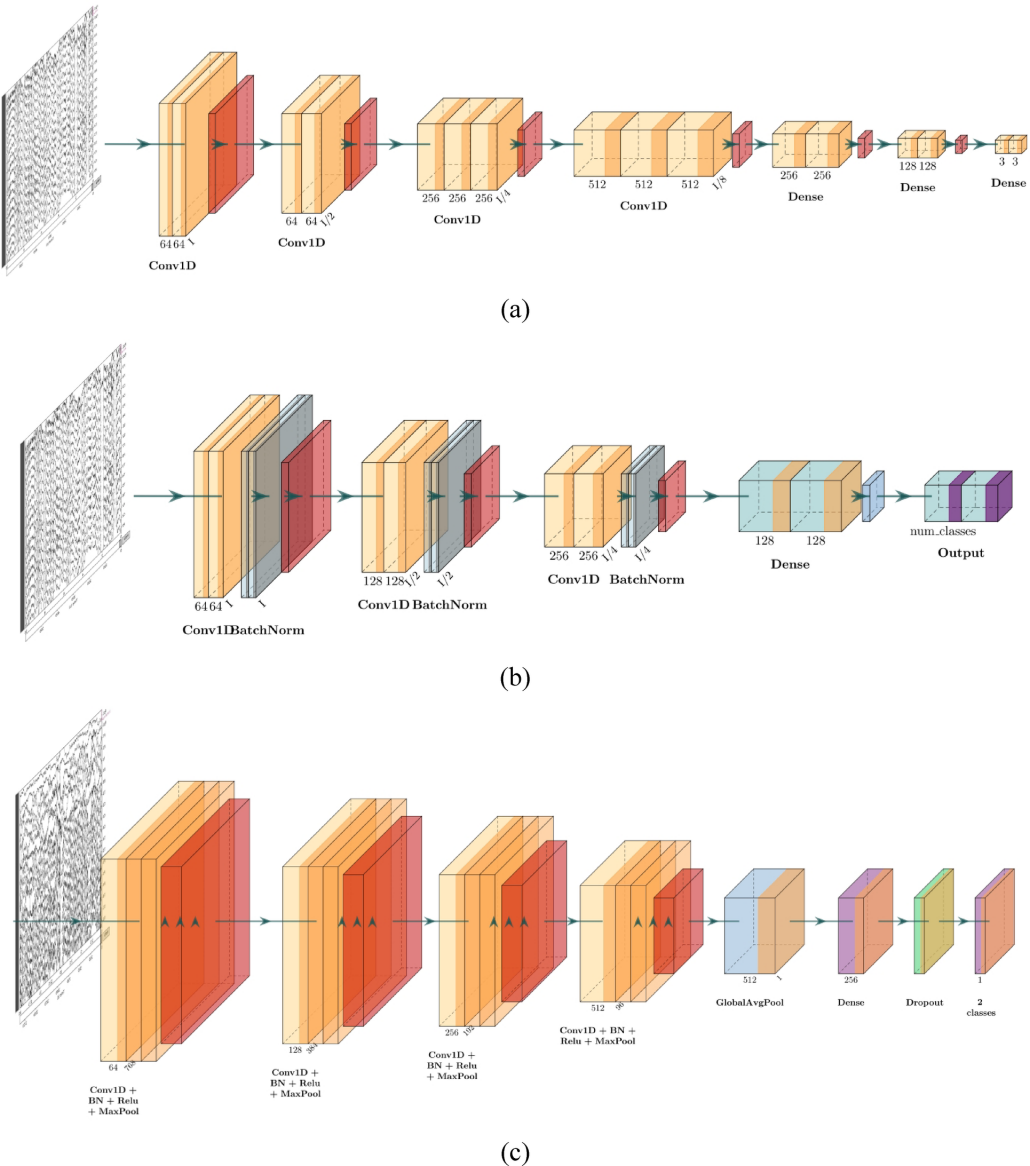
1D CNN model architecture: (**a**) Dataset A, (**b**) Dataset B, (**c**) Dataset C.

The pre-processed data is considered directly for classification using CNN models. Compared to case study A, where for each recording, PSD was computed from which suitable features were extracted and loaded to the ML model, in this case study, DL models are employed, wherein 19-channel pre-processed data is directly fed to CNN models. Additionally, instead of considering a generic model, CNN models are tailored for each dataset. Figure 3 enumerates the CNN architecture developed, and Table 4 depicts the hyperparameters obtained using grid search for the designed models.

**Table 4:** 1D CNN model hyperparameter details.

Hyperparameters	Model 1	Model 2	Model 3
Learning Rate	0.0001	0.001	0.001
Number of Epochs	10	20	20
Batch Size	32	32	32
Dropout Rate	0.5	0.5	0.5
Early Stopping Patience	3	–	10
L2 Regularization	–	–	0.001
Optimizer	Adam	Adam	Adam
Cross-validation	Group K fold (5)	Stratified K-fold	Group K fold (3)
Criterion	Cross Entropy Loss	Categorical Cross entropy	Binary Cross entropy
Kernel Size	3	3	3
Segmented Epoch	(196, 19, 2000)	(3796, 768, 19)	(16971, 768, 19)
Number of Channels (Conv)	32,64, 64	64, 128, 256	64, 128, 256, 512
Pooling Size	2	2	2

The implementation of 1D CNN involved loading segmented epochs. Cross-entropy was employed as the loss function, with dropout layers added to prevent overfitting. Early stopping was implemented based on validation accuracy. Post-training, the model’s performance was evaluated using various metrics, and learning dynamics were analysed through training and validation loss curves, as shown in the supplementary material. The model achieves a strong performance with Dataset A, reaching an accuracy of 90.00%. This aligns with the earlier observed trends of decreasing training and validation losses, indicating effective learning of the dataset’s patterns. In contrast, Dataset B achieves the highest accuracy at 98.74%, demonstrating exceptional model performance and a strong ability to generalize to new data. Although there were occasional spikes in validation loss, the model still performs exceptionally well on Dataset B. On the other hand, Dataset C shows a lower accuracy of 78.03%, reflecting significant challenges for the model. This reduced accuracy is in line with the previously noted erratic and increasing validation loss, suggesting issues with overfitting and poor generalization for two reasons: firstly, the signal is more complex than the other two datasets, and second, the data is not completely pre-processed. This analysis underscores the impact of dataset characteristics on model accuracy and the need for targeted adjustments, especially to improve performance on Dataset C.

#### Case study C

**Fig. 4:**
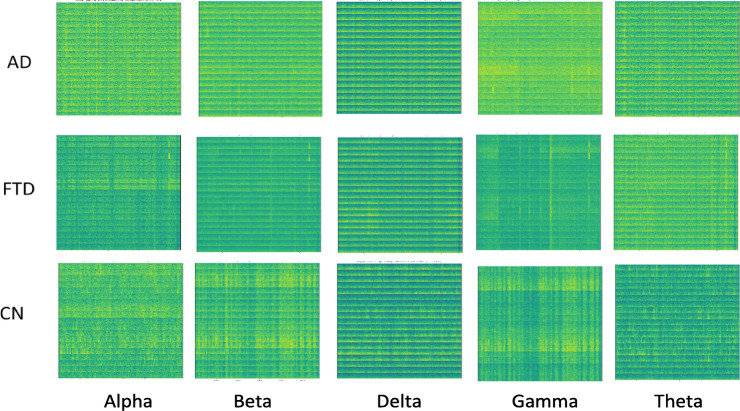
Illustration of spectrogram generated for 19 channel data for each band; Dataset A.

**Fig. 5:**
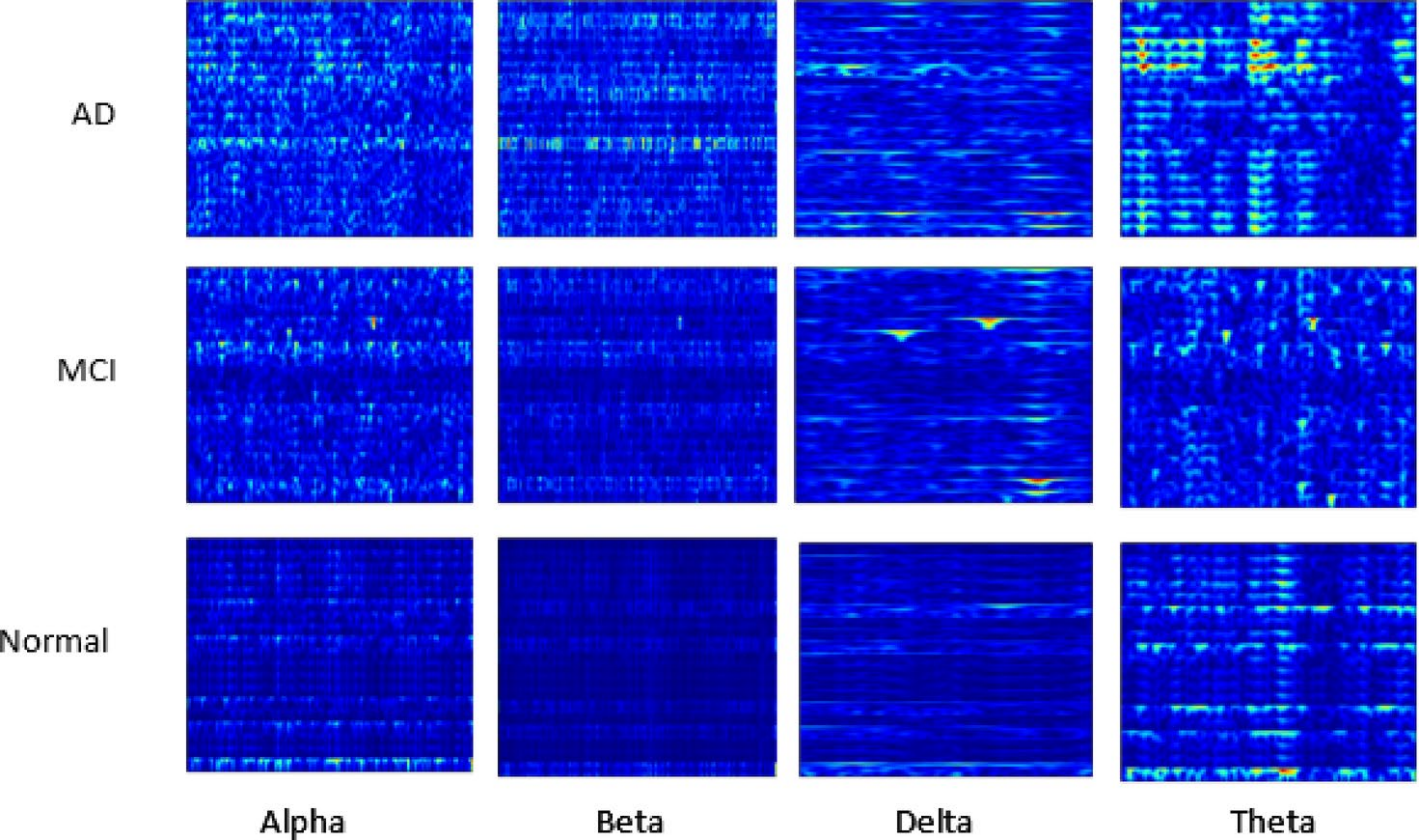
Illustration of spectrogram generated for 19 channel data for each band; Dataset B.

**Fig. 6:**
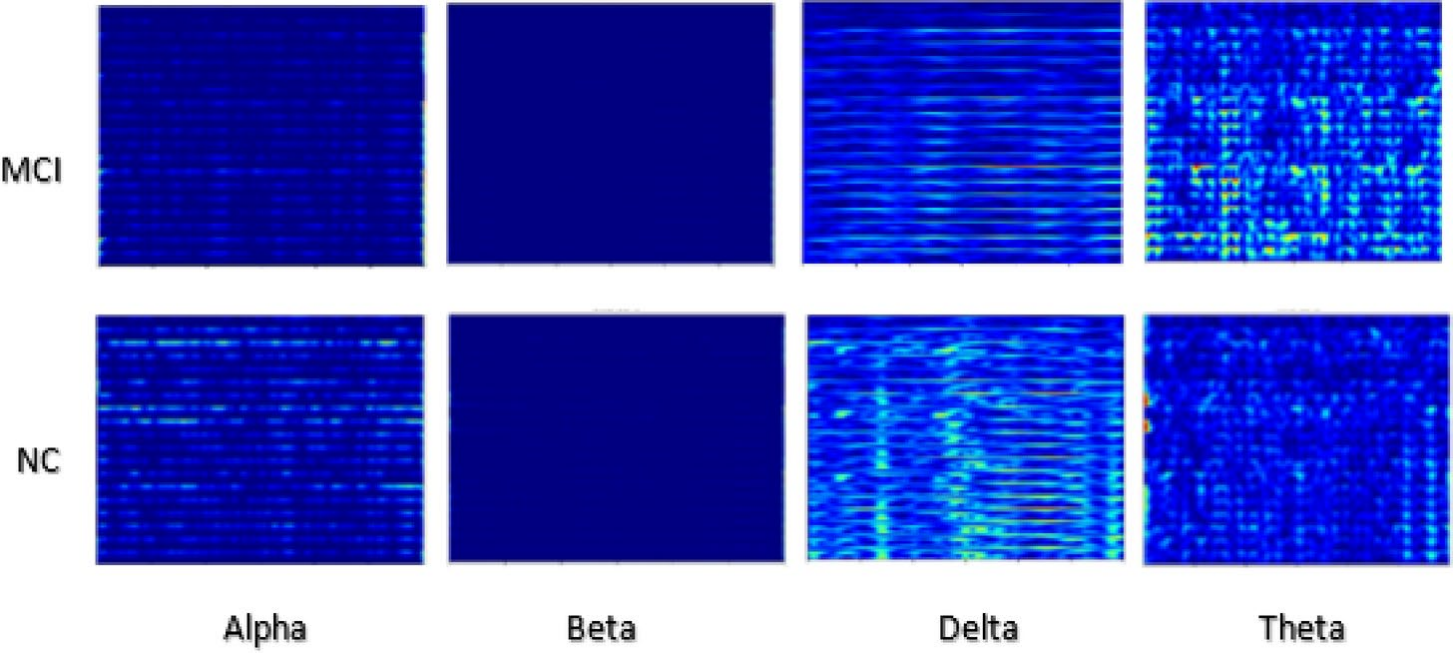
Illustration of spectrogram generated for 19 channel data for each band; Dataset C.

The 2D CNN model processes spectrograms generated from the EEG signals, learning to identify patterns in the time-frequency representation of the data (Figs. 4, 5 and 6). Each EEG channel underwent processing to compute Morlet wavelet transforms tailored to each frequency band. An epoch duration of 4 s was chosen, and the sampling rate from the loaded EEG information was used to define a time vector ‘t.’ A Morlet wavelet function was defined to model the time-domain wavelet, leveraging the central frequency of each band to ensure accurate transformation. For each channel, the wavelet transform was computed by convolving the EEG signal with the Morlet wavelet (Table 5). Subsequently, spectrograms were computed using the resulting wavelet decompositions, with parameters set to achieve optimal frequency resolution. Each spectrogram was then plotted and saved as an image (PNG) file, while the decomposed data was saved in NPZ format to facilitate further analysis. Further, all channel spectrograms were stacked for each frequency band, enabling the creation of comprehensive images representing the collective spectral power distribution across channels. Table 6 presents spectrogram results across datasets, with different degrees of spectral synchrony, signal complexity, power distribution, and EEG rhythm stability between AD, FTD, MCI, and CN groups. The findings suggest differential patterns of neural communication, with AD showing widespread impairment, MCI partial disruption, and CN normal functioning.

**Table 5:** 2D CNN model spectrogram generation details for all datasets.

Parameter	Details
Wavelet Transformation	Morlet wavelet transformation
Epoch Duration	19 channels 4 s, 3 s
Sampling Rate	500 Hz, 256 Hz
Segment Length	2000 samples (~ 4 s), 768 samples (~ 3 s)
Spectrogram Computation Method	Convolution of the EEG signal with the Morlet wavelet, then visualized as a color-coded image.
Number Spectrograms Generated	Dataset A: 440, Dataset B: 15,144, Dataset C: 64,800
Spectrograms Used for Model Input	Dataset A: 440, Dataset B: 4,320, Dataset C: 4,604
Frequency Ranges for Wavelet Scales	Dataset A: Delta, Theta, Alpha, Beta, Gamma Dataset B & C: Delta, Theta, Alpha, Beta
Spectrogram Visualization	Time on the x-axis Frequency on the y-axis Color bars to indicate power levels
Additional Information	Aggregated spectrograms across all channels for each frequency band Stacked spectrogram data to create comprehensive images of spectral power distribution across channels

**Table 6:** Observations of spectrogram images.

Feature	Dataset A	Dataset B	Dataset C
Spectral Synchrony	AD: Poor synchrony FTD: Moderate CN: High	AD: Highly disrupted MCI: Partial loss CN: Consistent	MCI: Partial disruption CN: High
Signal Complexity	AD: Reduced complexity FTD: Slight reduction CN: High	AD: Low complexity MCI: Moderate CN: High	MCI: Reduced complexity CN: Normal
Power Distribution	AD: Shift to lower bands FTD: Mild shift CN: Balanced	AD: Skewed towards delta/theta MCI: Shifted CN: Balanced	MCI: Shift towards slow bands CN: Balanced
EEG Rhythms Stability	AD: Irregular FTD: Mild disruption CN: Stable	AD: Highly irregular MCI: Partially disrupted CN: Consistent	MCI: Disrupted CN: Stable
Delta/Theta Dominance	AD: High dominance FTD: Moderate CN: Low	AD: Highly dominant MCI: Moderate CN: Minimal	MCI: Increased CN: Low
Beta/Alpha Reduction	AD: Severe reduction FTD: Mild reduction CN: Normal	AD: Severe loss MCI: Slight reduction CN: Balanced	MCI: Reduced CN: Normal
Clinical relevance-Neural Communication	AD: Impaired FTD: Partial CN: Normal	AD: Severely impaired MCI: Mild impairment CN: Normal	MCI: Partial impairment CN: Normal

Model performance was evaluated on three datasets (A, B, C) using four metrics: Accuracy, Precision, Recall, and F1-score. Dataset A excels with metrics around 91.10-91.92%, while Dataset C performs poorly with values near 66.00-66.20%. Dataset B yields good results, around 86.25-87.61%. The model is highly effective on A, moderately effective on C, and least effective on B. The characteristics of the datasets influence model performance, highlighting the importance of careful dataset selection. Model run times are as follows: A (GPU) 21 min, B (GPU) 37 min, C (CPU) 600 min.

The model’s classification performance for AD, FTD, and CN is examined with the confusion matrix, with 1523 and 746 accurate predictions, respectively; it correctly classifies AD and CN. It is difficult to differentiate this class, though, as FTD is commonly misclassified as AD or CN. The 746 samples of CN that were incorrectly classified as AD indicate feature overlap between these classes. The matrix for AD, MCI, and CN for dataset B, with 1533 and 309 accurate predictions, respectively, the model does well for AD and CN. However, MCI is frequently misclassified; overlapping features are highlighted by the fact that 233 samples are classified as AD and 35 as CN. In the MCI and CN classification results of dataset C, although the model correctly identifies 507 MCI and 923 CN samples, there is a significant amount of misclassification (387 MCI as CN and 343 CN as MCI), indicating overlapping features and the need for additional optimization to improve MCI and CN discrimination.

A Statistical test, ANOVA^12^, was implemented with and without replication, and a few observations were made. The datasets show meaningful variation, with an F-value far exceeding the critical value and a very small P-value (< 0.05). This suggests that the datasets are statistically different from each other. The performance metrics do not exhibit significant differences, as the P-value is far greater than 0.05 and the F-value is less than the critical value. Any observed differences in performance metrics are likely due to random variability. The lack of significant interaction implies that the differences in performance metrics are consistent across datasets.

The Wilcoxon Signed-Rank Test^27^ is also implemented on the evaluation metrics, where interesting, contradicting results are observed. The Wilcoxon test suggests that there is no significant difference between the models when comparing paired values across metrics. ANOVA examines variations in means among several models. Whereas, the Wilcoxon Examines variations in paired samples (median or ranks). ANOVA is more sensitive to mean differences. Wilcoxon concentrates on changes in distribution and is less sensitive to minute variations. Because the models’ performances are reasonably close, there are enough mean differences for ANOVA but no significant paired differences in Wilcoxon. Further, Tuckey’s HSD test^28^ is performed to identify the pairwise difference. All pairwise comparisons show statistically significant differences with p-values of zero. The differences between Model-1, Model-2, and Model-3 are statistically significant. These findings underscore the dataset differences but also suggest that the performance metrics are not a primary driver in the variability of the analysis.

## Discussion

This paper explores methods for diagnosing patients with dementia based on EEG signals utilizing three separate techniques. In the first approach, a traditional ML model was built after processing the data and extracting features from the PSD using an RF classifier. Secondly, a 1D CNN was proposed to take the pre-processed EEGs directly as input. Third, this work used the 2D CNN to classify the spectrogram images by inputting decomposed EEG signals as images.

The research shows that 1D and 2D convolution models improve classification performance compared to traditional ML models. The RF classifiers and SVMs used conventional approaches based on handcrafted features, which may fail to comprehend the complex spatiotemporal reliance in EEG signals. On the other hand, CNNs automatically discover hierarchical features from the raw EEG data, enhancing their capacity to recognize subtle neurological patterns.

The development of 1D and 2D CNN architectures enables the discovery of neurophysiological markers that are usually hard to obtain using conventional methods:

- 1D CNN Models: These models are well suited to capture temporal features from raw EEG signals, allowing detection of transient bursts of activity and loss of synchronization, two of the best early markers of neurodegenerative diseases. Moreover, subtle changes in PSD and phase synchronization among EEG channels signify alterations in brain connectivity related to cognitive impairment.
- 2D CNN Models: 2D CNNs extract complex spatiotemporal patterns from the topographical maps or spectrograms generated from EEG signals, which are essential for diagnosing dementia. These models are based on the analysis of time–frequency dynamics of electrophysiological signals and allow the visualization of characteristic dynamics reflecting the stage of dementia. The spatial distribution of neural activity is also investigated, aiding in the identification of spatial cognitive deficits associated with functional impairment in dementia.

This study performs binary (2-class) and multi-class (3-class) classifications, using ML, 1D DL, and 2D DL models. The findings yield important insights in the diagnosis of neurodegenerative disease by identifying temporal deviations, spatial-temporal correlations, and frequency band power distributions predictive of cognitive impairment. The insights would help improve diagnostic tools, facilitate early detection, accurate classification, and staging of the disease to enable well-informed intervention planning.

Important directions for improvement include advanced artifact rejection methods like ASR or Surface Laplacian Filtering, optimizing feature extraction techniques, improving model generalization on different datasets, and combining multimodal data sources such as EEG and MRI. Future studies may also investigate adaptive DL architectures that can dynamically adapt to individual patient characteristics and further improve clinical utility.

The study evaluates three benchmark datasets with class labels of cognitively normal FTD patients, MCI cases, and Alzheimer’s disease subjects. The retrospective comparison between these datasets illustrates that DL models, especially 1D and 2D CNNs, consistently outperformed traditional methods in identifying specific EEG features associated with neurodegenerative disease pathology.

DL models are more powerful, but at the cost of huge computational overhead, thus making them hard to deploy on commodity hardware. Improving the efficiency of the model makes the practical realization feasible, and the effective utilization of the high-performance GPU platform can also effectively improve the model performance. Furthermore, DL models lack interpretability, which might limit their trust and adoption in clinical practice. By incorporating various Explainable AI (XAI) techniques throughout the learning process, we can enhance the transparency of the algorithm, allow for insights into how the model makes decisions, and allow for more integration into clinical practice.

## Conclusion and future work

This work has identified the hidden pattern for each dementia, considering both across PSD and with spectrogram images. Clinical relevancies are derived from the fundamentals, which are the necessity for State-of-the-Art model development in diagnosing different stages/types of dementia. Practical limitations/challenges are identified across three different datasets, starting from pre-processing to model tuning. Generalizability of the model across datasets is investigated. However, a statistical difference was found across datasets and models. The random forest model considered handcrafted features from spectra in the bands of alpha, beta, gamma, theta, and delta. Although it performed well, SHAP analysis assisted in realizing the existence of a specific relevant feature for a particular class. Further, the implementation of 1D CNN explored the possibilities of diagnosing the EEG state directly from raw EEG data. However, the 1D CNN model results in better performance compared to the 2D CNN models. Strength of Alzheimer’s disease multi-class classification based on 1D and 2D CNN models based on the persisting results in tables has been evidently shown in this study, especially offering outstanding results from 1D CNN on Dataset B and between training and validation data, maintaining good generalization. Again, Dataset A displayed balanced models as the 1D and 2D CNN models outperformed those in reference studies, showing that they learned and generalized. However, Dataset C posed challenges; the 2D CNN was faced with overfitting and weak generalization. Although these are encouraging findings, there are limitations to the study. As a result of restricted computational resources, a lower number of cross-validation folds and training epochs were considered, collectively possibly leading to sub-optimal models. Data imbalance further complicated the training process. Despite the implementation of band-pass filtering and ICA steps during pre-processing, the scope of the analysis could have been expanded further by exploring additional parameters. Additionally, the heterogeneity of the datasets, two of which contain three class labels and one which contains two labels, may introduce inconsistencies that impact the model’s generalizability. Statistical tests ANOVA and Wilcoxon contradicted in their results, which led to an investigation by Tuckey’s HSD test.

Future works can be extended to enhance model performance, generalizability, and interpretability in EEG-based classification techniques. Exploration of SMOTE and GAN-based augmentation to balance classes, further improve the robustness of the model. Advanced transformer architecture can be implemented to capture long-range dependencies in EEG signals efficiently. Also, hybrid models taking advantage of both 1D and 2D CNNs will be optimized for better spatial and temporal feature extraction. Sophisticated methods, such as attention mechanisms and graph neural networks, can be explored to improve classification accuracy.

## Electronic supplementary material

Below is the link to the electronic supplementary material.

Supplementary Material 1

## Data Availability

The datasets used in this work are available at:  https://misp.mui.ac.ir/en/eeg-data-0; https://figshare.com/articles/dataset/dataset_zip/5450293?file=9423433; https://openneuro.org/datasets/ds004504/versions/1.0.8.
