# Supplementary material for: EEG-based neurodegenerative disease diagnosis: comparative analysis of conventional methods and deep learning models
Source: Sci Rep. 2025 May 7;15:15950. doi: 10.1038/s41598-025-00292-z (PMC12058994; doi:10.1038/s41598-025-00292-z)
Supplement: Supplementary file 1 — Supplementary Material 1 [file 41598_2025_292_MOESM1_ESM.docx]

**Supplementary Material**

**Dataset A** was selected based on OpenNeuro, a validated dataset with complete and relevant features for AD-related studies. EEG resting state data are available for 88 subjects, grouped into three categories (AD, n = 36), (FTD, n = 23), and (CN, n = 29). The recording was performed using 19 scalp electrodes according to a 10–20 international system with extensive sampling at 500 Hz and 10 µV/mm resolution. Total recording periods achieved in the dataset are considerable, that is, 485.5 minutes for AD, 276.5 minutes for FTD, and 402 minutes for CN, with AD patients achieving on average 13.5 minutes, FTD 12 minutes, and CN 13.8 minutes. Participants’ cognitive and neuropsychological conditions were assessed using the Mini-Mental State Examination (MMSE), yielding average scores of 17.75 (SD = 4.5) for AD, 22.17 (SD = 8.22) for FTD, and 30 for CN, indicating a clear gradient of cognitive decline. The median disease duration for the AD group was 25 months, with an interquartile range of 24 to 28.5 months. No dementia-related comorbidities were reported for the AD group. Although specific information regarding gender and ethnicity is not included, the dataset adheres to GDPR guidelines, ensuring the anonymization of personal data. The data was collected at the 2nd Department of Neurology at AHEPA General Hospital in Thessaloniki, a reputable institution known for its focus on neurological disorders, which adds credibility and standardization to the dataset. The comprehensive clinical and demographic characteristics of this dataset make it a valuable resource for investigating EEG biomarkers in AD and related conditions.

**Dataset B** is believed to originate from the Figshare website. EEG data were obtained from 59 patients with moderate dementia, seven patients with mild cognitive impairment (MCI), and two controls. The EEG data is stored as *.mat files. Within this dataset, there are 33 AD patients and 4 MCI patients, whose data were collected at a sampling frequency of 256 Hz, and two normal controls with a sampling frequency of 128 Hz. The average MMSE score for the AD group was 14.9 (standard deviation = 2.3). The mean ages of all three groups were 70.5 ± 4.9 years in the AD group, 67 ± 7.6 years in the MCI group, and 72.2 ± 5.3 years for the normal subjects. The signals were recorded using a Digital EEG setup (Walter EEG PL-231, Germany) with a TruScan 32 (Alien Technik Ltd., Czech Republic).

The selection of the **Dataset C** EEG Signals from Normal and MCI provides valuable insights into cognitive impairment research with EEG recordings from 27 participants, categorized into MCI with 11 individuals and NC with 16 individuals. The average age of the MCI group is 66.4 ± 4.6 years, while the NC group averages 65.3 ± 3.9 years. Cognitive assessments were conducted using the MMSE, with scores ranging from 21-26 for the MCI group and >26 for the NC group. Confirmation of diagnosis was achieved using the Neuropsychiatry Unit Cognitive Assessment Tool (NUCOG), where scores of 86.5 indicate normal cognitive function and 75-86.5 denote MCI. The recordings were collected in 2015 from the cardiac catheterization units of Sina and Nour Hospitals in Isfahan, Iran, using a 32-channel digital EEG device (Galileo NT, EBneuro, Italy) with 19 scalp electrodes. Figure 1 below presents the dataset recordings accessed. These are the figures showing the channels and classes for this dataset: 5 out of 19 channels are related to each class. Dataset C, which is acquired, is in its raw state.


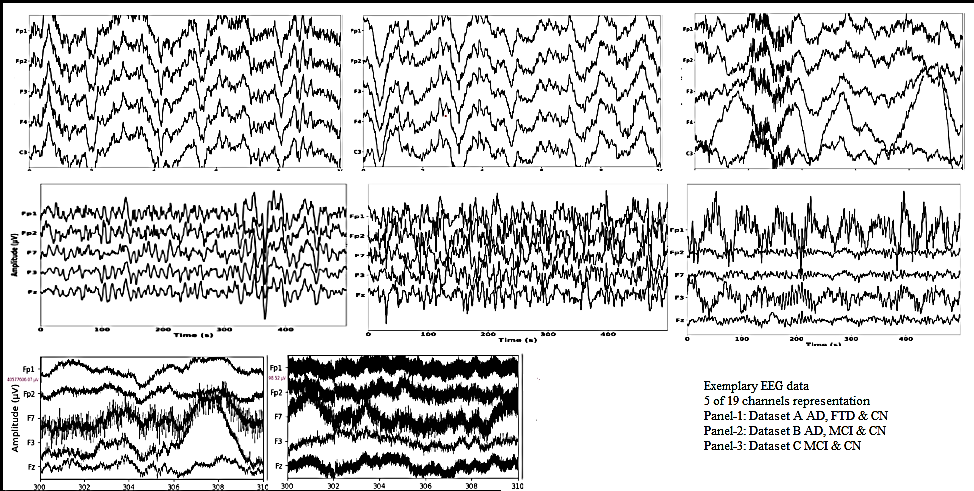


**Sample EEG data from datasets A, B, and C**


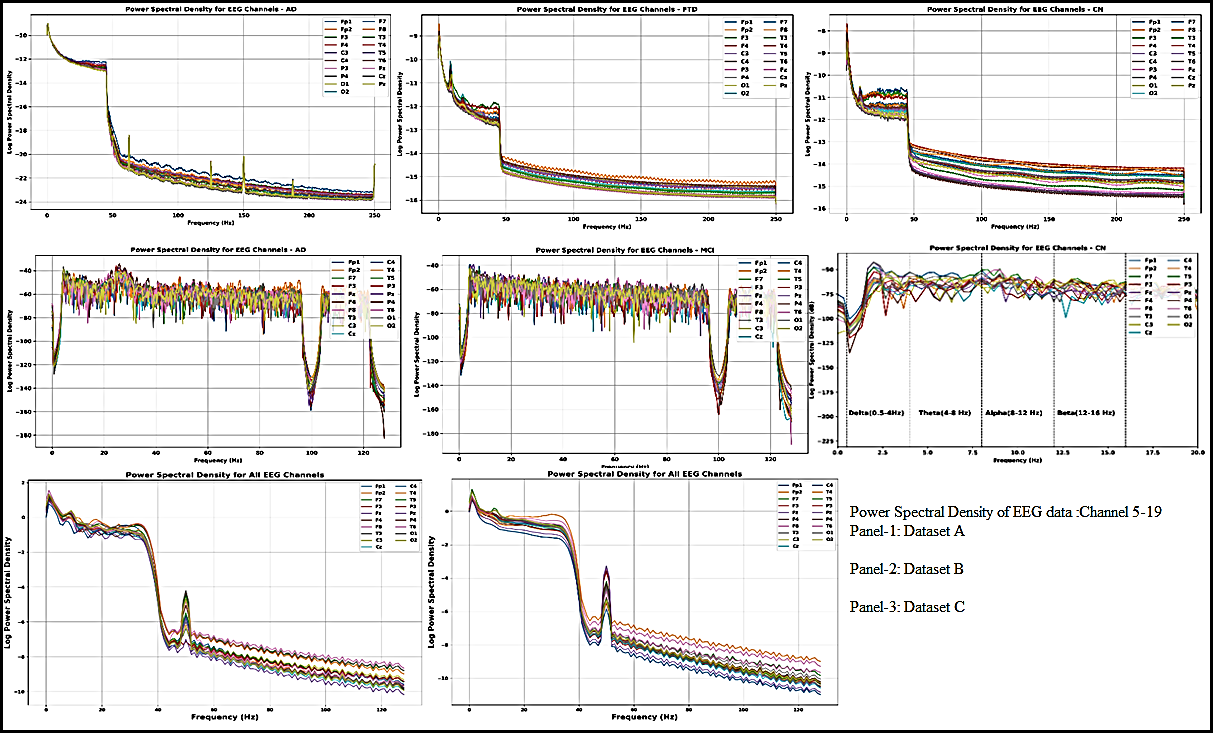


**Power Spectral Density (PSD) of 19 channels of EEG data**

**
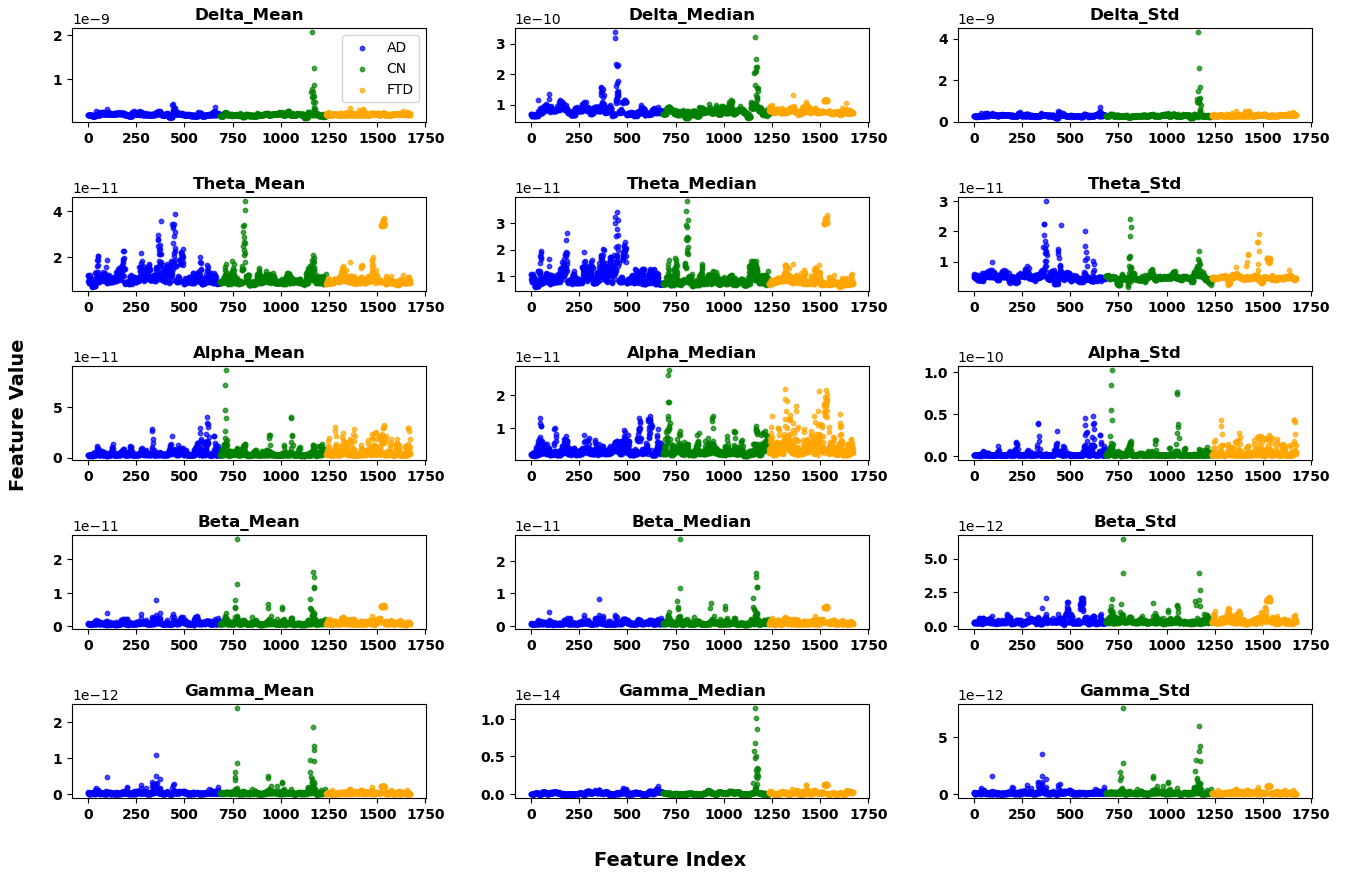
**

**Scatter plot showcasing the distribution of EEG signal features (Mean, Median, and Standard Deviation) across frequency bands (Delta, Theta, Alpha, Beta, Gamma) for AD, CN, and FTD groups.**


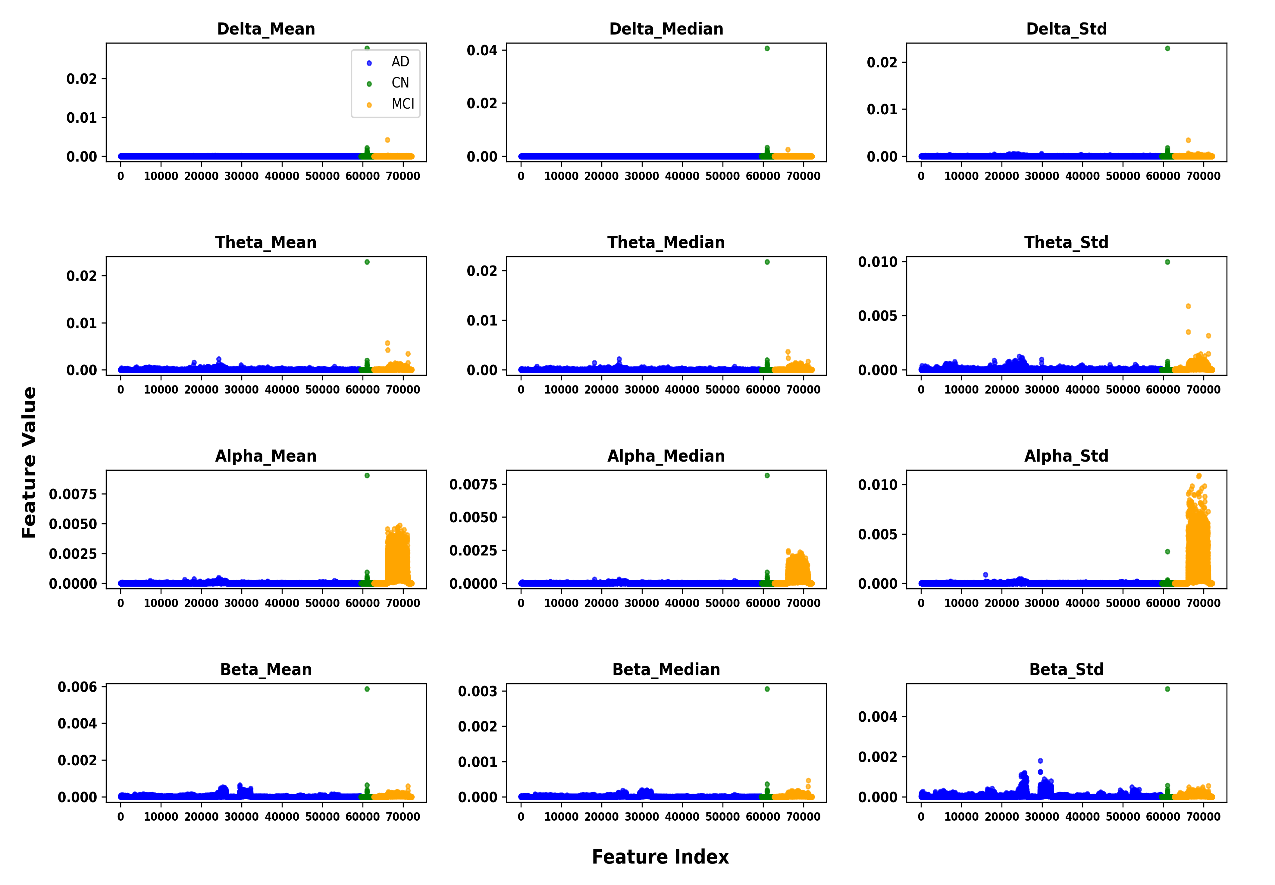


**Scatter plot showcasing the distribution of EEG signal features (Mean, Median, and Standard Deviation) across frequency bands (Delta, Theta, Alpha, Beta) for AD, MCI, and CN groups**


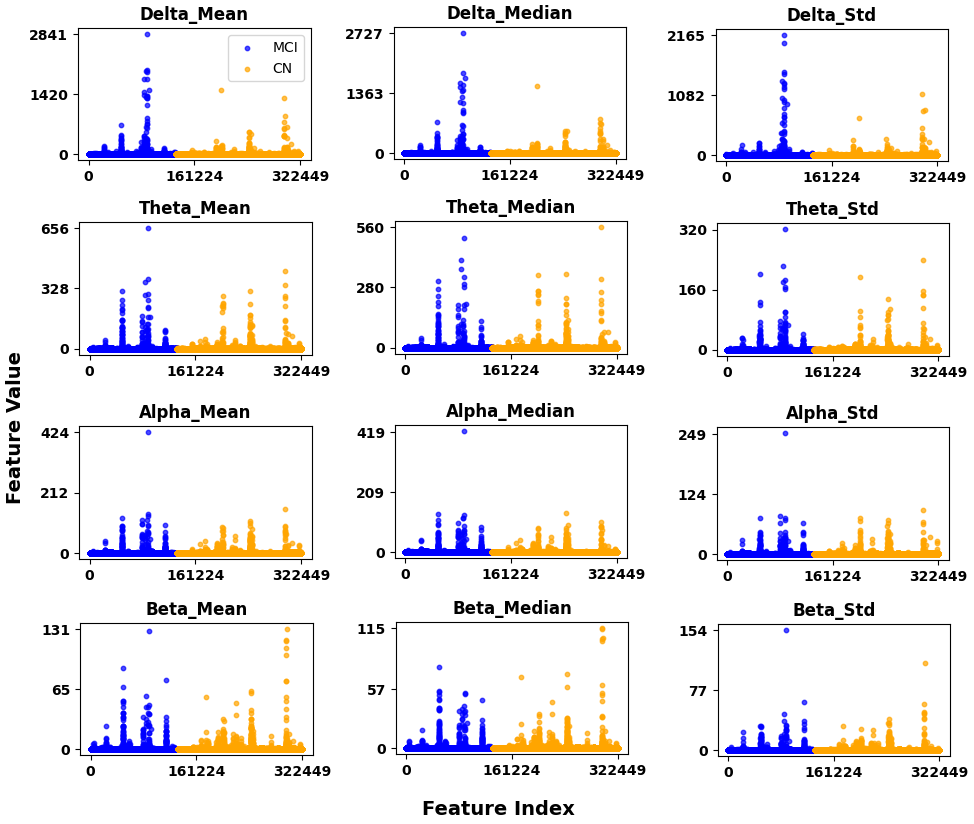


**Scatter plot showcasing the distribution of EEG signal features (Mean, Median, and Standard Deviation) across frequency bands (Delta, Theta, Alpha, Beta) for MCI and CN groups**


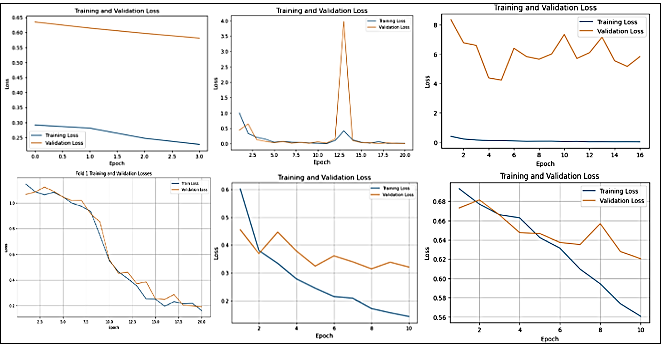


**Training and Validation Loss Plot for 1D CNN and 2D CNN for datasets A, B, and C**


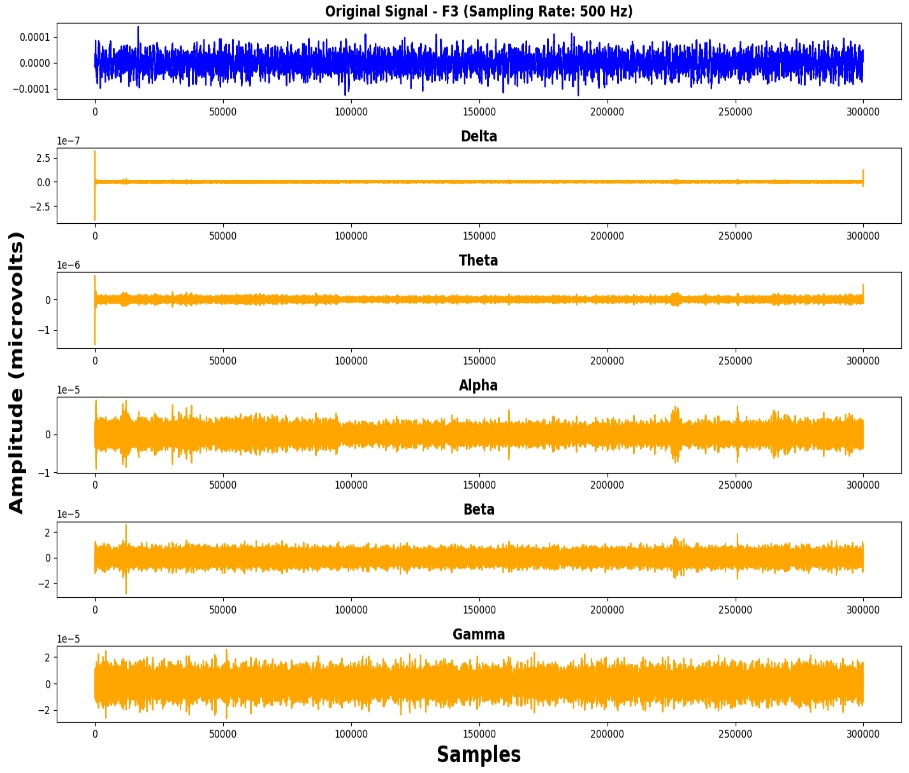


**Visualization of EEG Signal and Frequency Bands: The top panel shows the original EEG signal recorded at the F3 channel (sampling rate: 500 Hz). Subsequent panels depict the decomposed frequency bands (Delta, Theta, Alpha, Beta, Gamma), highlighting the respective amplitude variations across samples.**
